# The role of ferroptosis‐related genes for overall survival prediction in breast cancer

**DOI:** 10.1002/jcla.24094

**Published:** 2021-11-06

**Authors:** Li‐Yan Jin, Yan‐lin Gu, Qi Zhu, Xiao‐hua Li, Guo‐Qin Jiang

**Affiliations:** ^1^ Department of Thyroid and breast surgery The Second Affiliated Hospital of Soochow University Suzhou China; ^2^ Department of Thyroid and breast surgery Traditional Chinese medicine hospital of kunshan Kunshan China; ^3^ Department of Thyroid and breast surgery Wuzhong People's Hospital of Suzhou City Suzhou China

**Keywords:** bioinformatic analysis, biomarker, breast cancer, ferroptosis, overall survival

## Abstract

**Background:**

Ferroptosis is a novel iron‐dependent form of cell death, which is implicated in various diseases including cancers. However, the influence of ferroptosis‐related genes on the prognosis of breast cancer remains unclear.

**Methods:**

RNA sequencing data of 1053 breast cancer tissue samples and 111 normal tissue samples from The Cancer Genome Atlas (TCGA) were analyzed. Expression levels of 259 ferroptosis‐related genes were compared. Gene Ontology (GO) and the Kyoto Gene and Genomic Encyclopedia (KEGG) analyses were conducted on differentially expressed genes. Cox univariate analysis was conducted to explore the potential prognostic biomarkers of breast cancer. Infiltrating immune cell status was assessed.

**Results:**

A total of 66 ferroptosis‐related genes were differentially expressed in breast cancer tissues. The enriched GO terms included Biological Process (mainly included response to oxidative stress, cellular response to chemical stress, multicellular organismal homeostasis, cofactor metabolic process, response to metal ion, response to steroid hormone, cellular response to oxidative stress, transition metal ion homeostasis, iron ion homeostasis, and cellular iron ion homeostasis), Cellular Component (mainly included apical plasma membrane, early endosome, apical part of cell, lipid droplet, basolateral plasma membrane, blood microparticle, clathrin‐coated pit, caveola, astrocyte projection, and pronucleus) and Molecular Function (mainly included iron ion binding, ubiquitin protein ligase binding, oxidoreductase activity, acting on paired donors, with incorporation or reduction of molecular oxygen, oxidoreductase activity, acting on the CH−OH group of donors, NAD or NADP as acceptor, ferric iron binding, aldo−keto reductase (NADP) activity, oxidoreductase activity, acting on single donors with incorporation of molecular oxygen, steroid dehydrogenase activity, alditol:NADP+1−oxidoreductase activity, and alcohol dehydrogenase (NADP+) activity). The enriched KEGG pathway mainly included the HIF‐1 signaling pathway, NOD‐like receptor signaling pathway, ferroptosis, IL‐17 signaling pathway, central carbon metabolism in cancer, PPAR signaling pathway, PD‐L1 expression, and PD‐1 checkpoint pathway in cancer. Among them, 38 ferroptosis‐related genes were significantly associated with the prognosis of breast cancer. The prognostic model was constructed, and breast cancer patients in low‐risk group had a better prognosis. In addition, risk score of ferroptosis prognostic model was negatively correlated with B cells (*r* = −0.063, *p* = 0.049), CD8+ T cells (*r* = −0.083, *p* = 0.010), CD4+ T cells (*r* = −0.097, *p* = 0.002), neutrophils (*r* = −0.068, *p* = 0.033), and dendritic cells (*r* = 0.088, *p* = 0.006).

**Conclusions:**

The ferroptosis pathway plays a key role in breast cancer. Some differentially expressed ferroptosis‐related genes can be used as prognostic biomarkers for breast cancer.

## Graphical abstract

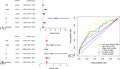

## 1 INTRODUCTION

Ferroptosis, an iron‐dependent form of nonapoptotic cell death, was first defined in 2012.^1^ It is caused by a dysfunction of oxidized membrane lipids, which reduces the activity of glutathione peroxidase 4. These contribute to the accumulation of lipid‐reactive oxygen species and induce cell death.^2^ Compared with non‐cancerous cells, cancer cells exhibit an increased iron demand.^3^ This is a potential promising method to treat cancer. Erastin was discovered to induce ferroptosis^4^ and can enhance the sensitivity of chemotherapy drugs, such as temozolomide^5^ and cisplatin.^6^

Breast cancer is the most common diagnosed cancer in women. There are an estimated 1.6 million cases of breast cancer each year with an approximately 6.4% mortality rate.^7^, ^8^ According to immunohistochemistry markers, breast cancer is divided into four subtypes (luminal A, luminal B, HER‐2‐enriched, and triple‐negative). Different subtypes of breast cancer require different therapies. Tumor staging, lymph node status, histological grades, and age are prognostic factors for breast cancer patients. Ma et al.^9^ reported that siramesine and lapatinib induced breast cancer cell death through ferroptosis, and after treatment with a ferroptosis inhibitor, breast cancer cell death was inhibited following the siramesine and lapatinib treatment. This indicates that the ferroptosis pathway plays an important role in breast cancer cells by treating with a lysosome disruptor and a tyrosine kinase inhibitor. We researched this article to gain insight into the potential clinical utility of ferritinophagy‐related genes, and we investigated the expression levels of ferritinophagy‐related genes in breast cancer and the molecular subtypes that contribute to the heterogeneity of breast cancer. Finally, we determined the relationships between ferroptosis and the prognosis of different subtypes.

The data of breast cancer patients were downloaded from The Cancer Genome Atlas (TCGA) database. The expression levels of ferritinophagy‐related genes were compared, and the prognostic model associated with the ferritinophagy‐related genes was constructed. We demonstrated that ferritinophagy‐related genes were crucial in the prognosis of breast cancer.

## 2 MATERIALS AND METHODS

### 2.1 Datasets and study cohorts

We downloaded the data of 1053 breast cancer patients and 111 normal tissue samples from the TCGA database. The RNA‐seq transcriptome data were matched to clinical pathologic information and were transformed into sample IDs by Perl. All patients included in this study were followed up for at least one month. Any incomplete survival information was excluded. A total of 259 ferritinophagy‐related genes were selected for subsequent bioinformatics analysis.

### 2.2 Bioinformatic analysis and statistical analysis

Differentially expressed genes (DEGs) from ferritinophagy‐related genes were analyzed between cancer tissues and normal tissues by the Wilcoxon test using the R v4.0.2. (http://www.r‐project.org/) with a cutoff criterion of *p* < 0.05. Gene Ontology (GO), and the Kyoto Encyclopedia Gene and Genomic (KEGG) analyses were conducted according to the DEGs. We performed the Cox univariate analyses to explore any genes associated with prognosis. Each prognosis‐related gene was given a risk score. Ferritinophagy‐related genes related to prognostic models were constructed through risk scores, and all patients were divided into two groups based on the risk scores. A Kaplan‐Meier plot was drawn to assess the survival differences between the two groups. The Cox univariate and multivariate analyses were performed to compare the relationship between clinicopathological variables and risk scores. The ROC curves were graphed to compare clinicopathological variables. To analyze tumor‐infiltrating immune cells, the data from the Tumor Immune Estimation Resource (TIMER) database were obtained. The risk scores for the ferritinophagy prognostic model and its correlation with immune infiltrates (B cells, CD4+ T cells, CD8+ T cells, neutrophils, macrophages, and dendritic cells) were analyzed.

## 3 RESULTS

### 3.1 Identifying differential expressions of ferritinophagy‐related genes

**FIGURE 1:**
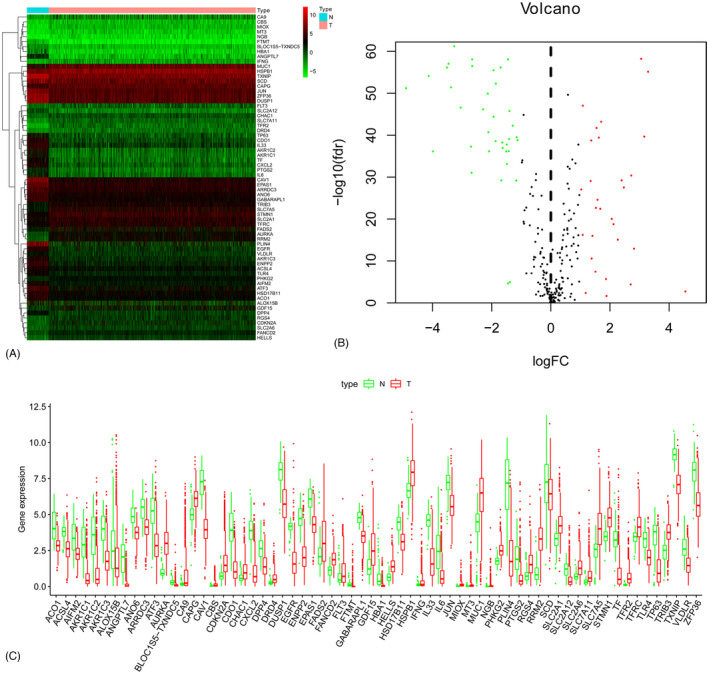
(A) Expression heatmap of differentially expressed ferroptosis‐related genes. (B) Expression volcano map of differentially expressed ferroptosis‐related genes. (C) Expression patterns of ferroptosis‐related genes in breast cancer types and paired non‐tumor samples

A total of 1053 breast cancer tissue samples (including 232 luminal A, 336 luminal B, 134 triple‐negative, and 351 HER‐2‐positive tissue samples) were included, and 49 patients were followed for less than 1 month. The expressions of 259 ferritinophagy‐related genes were compared between cancer tissue and normal tissue, and 66 genes were DEGs (FDR < 0.05 and log2 (fold change)> 1). The heatmap, volcano map, and boxplot of DEGs are shown in Figure 1.

### 3.2 Functional annotations of the differentially expressed ferritinophagy‐related genes

**FIGURE 2:**
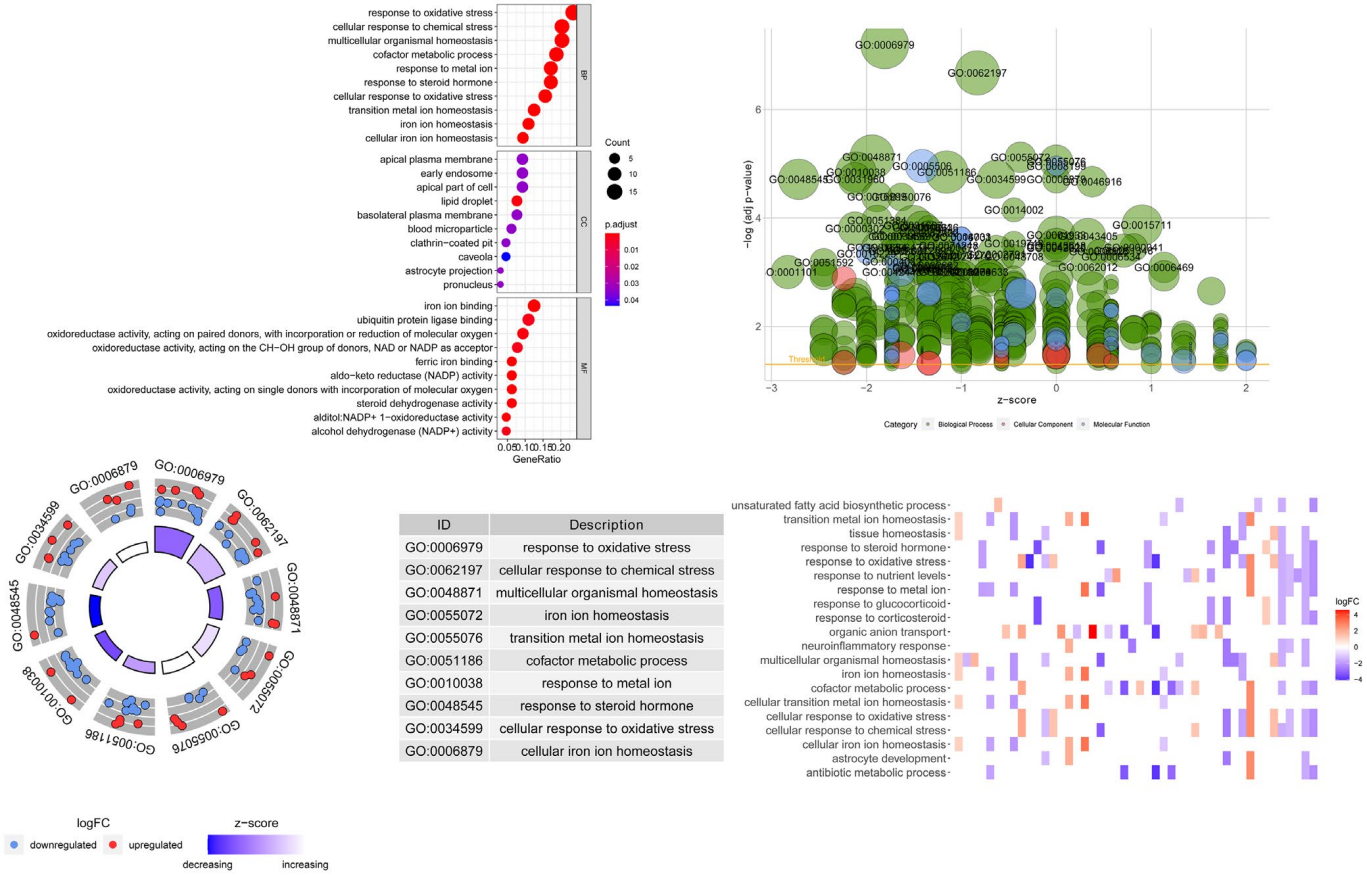
GO analysis of differential genes, including molecular function, cell component, and biological processes

The functional annotation analysis of the 66 DEGs provides a biological understanding for these genes. The top 10 of the three different GO levels (including molecular functions, cell components, and biological processes) are shown in Figure 2.

**FIGURE 3:**
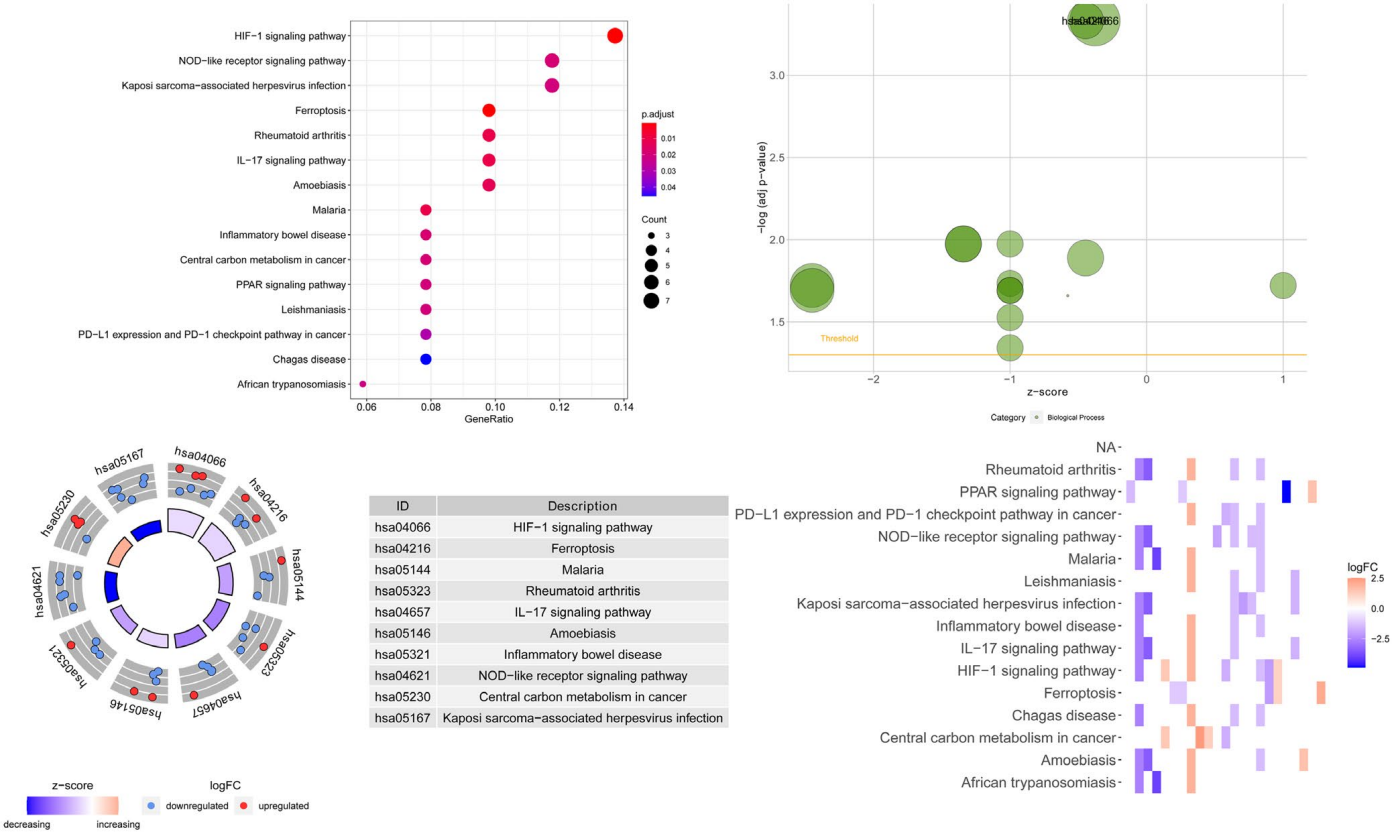
KEGG pathway enrichment analysis of differential genes

The enrichment analysis results of the top 10 main KEGG pathways are shown in Figure 3. These genes are closely related to the HIF‐1 signaling pathway, ferroptosis, IL‐17 signaling pathway, central carbon metabolism in cancer, PPAR signaling pathway, PD‐L1 expression, and PD‐1 checkpoint pathway in cancer, etc.

### 3.3 Identifying genes associated with prognosis

**FIGURE 4:**
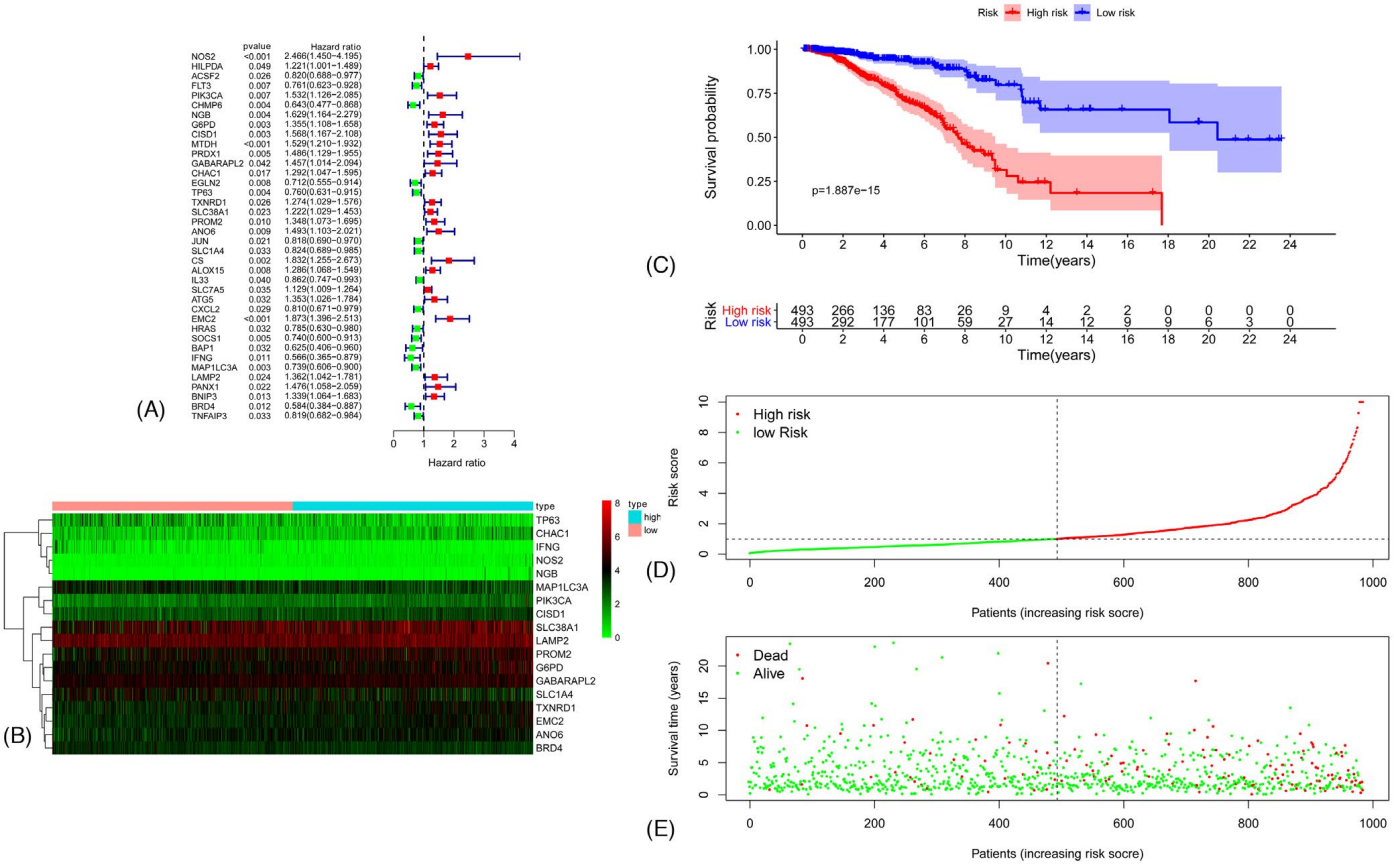
(A) Risk ratio forest plot showed the prognostic value of the gene. (B) The heatmap of prognostic gene. (C) Kaplan‐Meier Curve of high‐risk group and low‐risk groups according to the risk score. (D) Distribution of prognostic index. (E) Survival status of patients in different groups

The Cox univariate analysis was conducted to explore the potential genes associated with prognosis among 259 ferritinophagy‐related genes, and 38 genes had a close relationship with the prognosis (Figure 4A), while a total of 18 DEGs were significantly associated with prognosis. The expressions of these 18 DEGs are shown in Figure 4B. According to the Cox regression analysis, each prognosis‐related gene attained a risk score. A prognostic model was constructed through risk scores, and all patients were divided into two groups for overall survival based on the risk scores. Kaplan‐Meier plot is shown in Figure 4C. The patients in the low‐risk group had better prognosis than those in the low‐risk group. The distributions of the prognostic index and survival statuses of patients in the two groups are shown in Figure 4D, E, and the breast cancer subtypes were widely different. Genes associated with prognosis were analyzed, and the results are shown in [Link], [Link], [Link], [Link].

**FIGURE 5:**
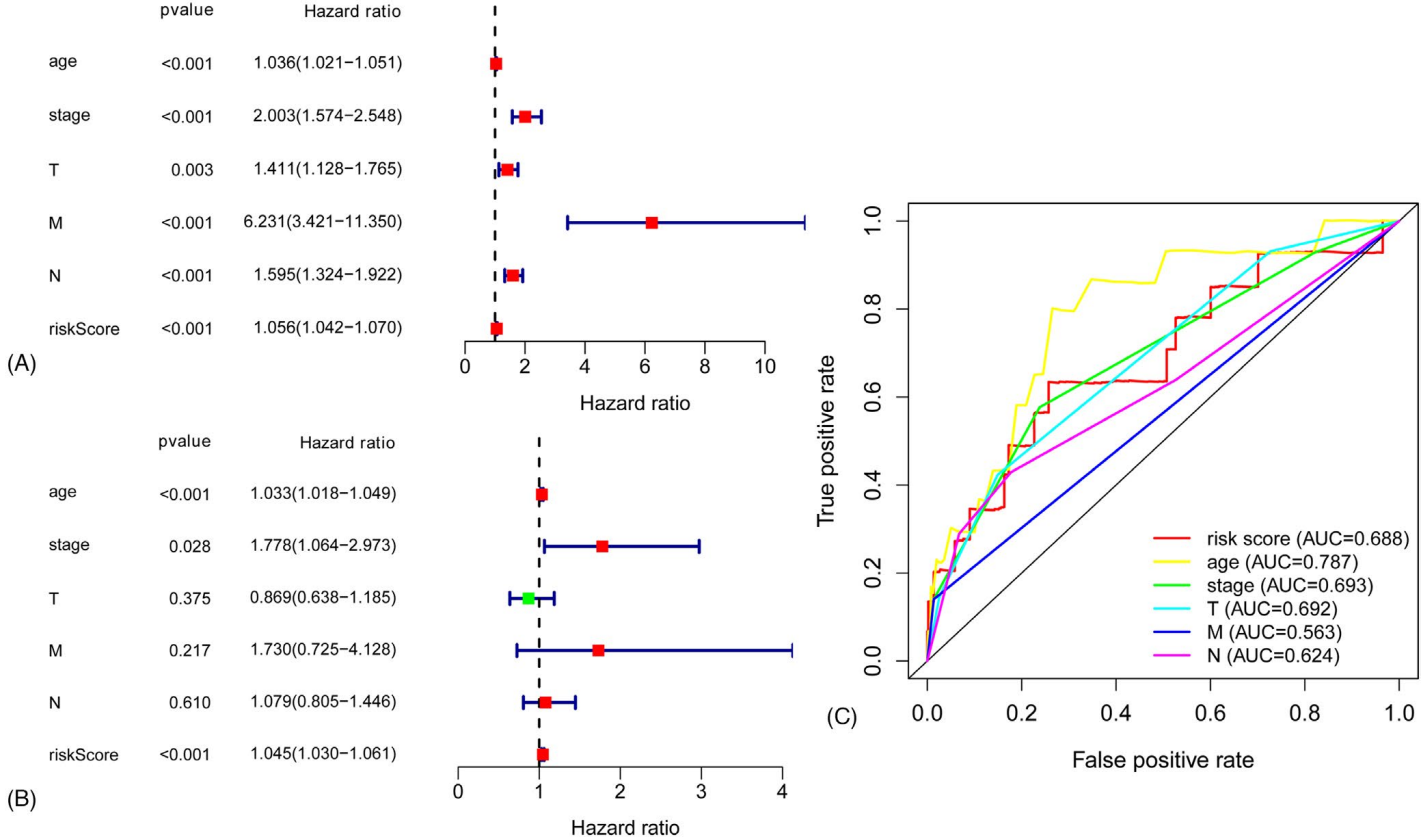
(A) A forest plot of Cox univariate analysis. (B) A forest plot of Cox multivariate analysis. (C) Survival‐dependent receiver operating characteristic curves for risk score, age, tumor size, and lymph node statue and metastasis statue

The Cox univariate analysis showed that the risk scores of ferritinophagy‐related genes, age, tumor size, and lymph node status and metastasis status show significant association with patient prognosis (Figure 5A). Cox multivariate analysis is shown in Figure 5B. The areas under the curve of the corresponding receiver operating characteristic curve for risk score, age, tumor size, and lymph node and metastasis statuses were 0.688, 0.787, 0.692, 0.624, and 0.563. They indicate that the prognostic index exhibited a certain potential prediction (Figure 5C).

**FIGURE 6:**
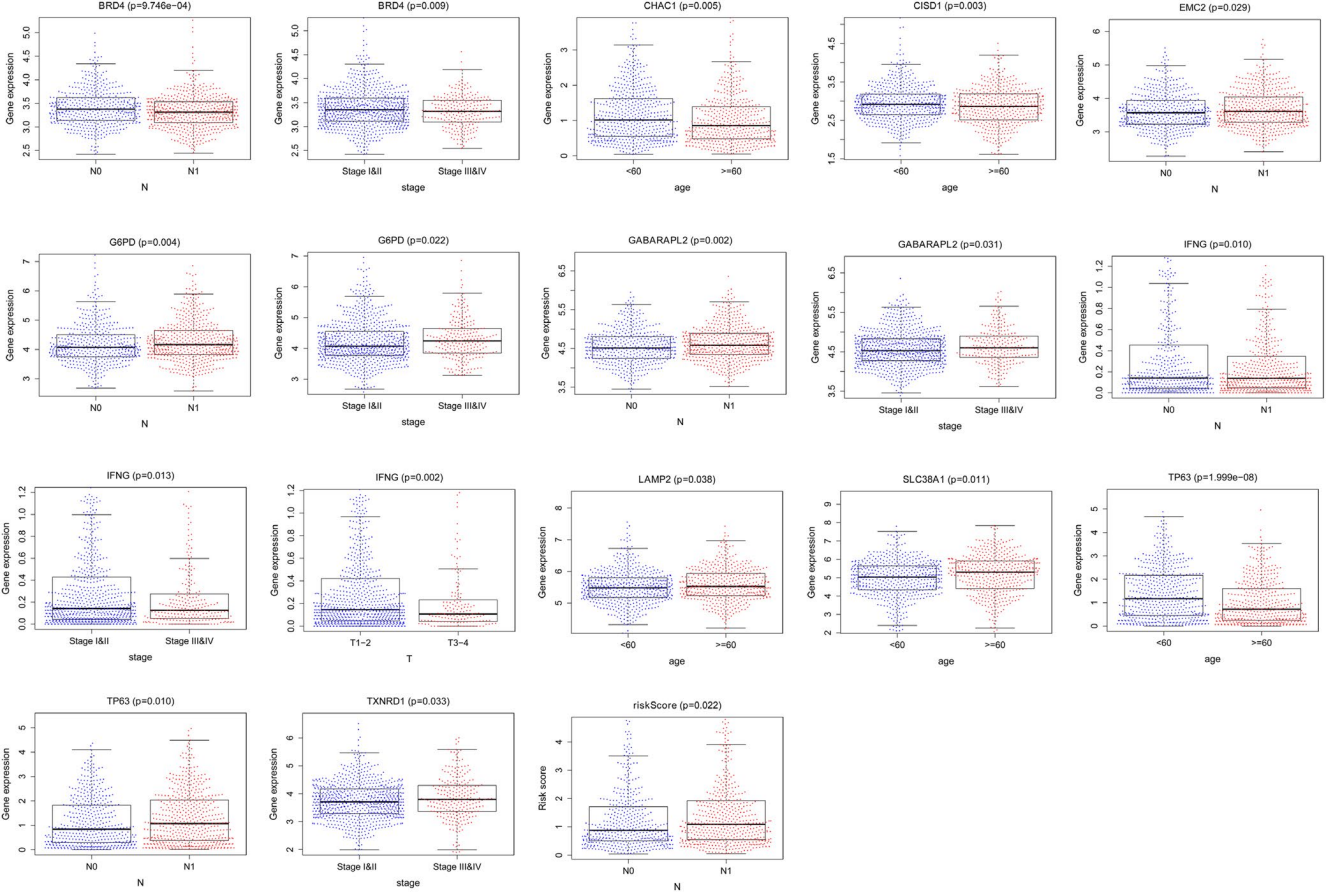
Clinicopathological features of the potential prognostic genes of breast cancer

The Wilcoxon test was conducted to compare the relationships between the clinical features (primary tumor status, lymph node status, age, stage, and risk scores) and the expressions of 18 potential prognostic genes. The results are shown in Figure 6.

### 3.4 Tumor‐infiltrating immune cells

**FIGURE 7:**
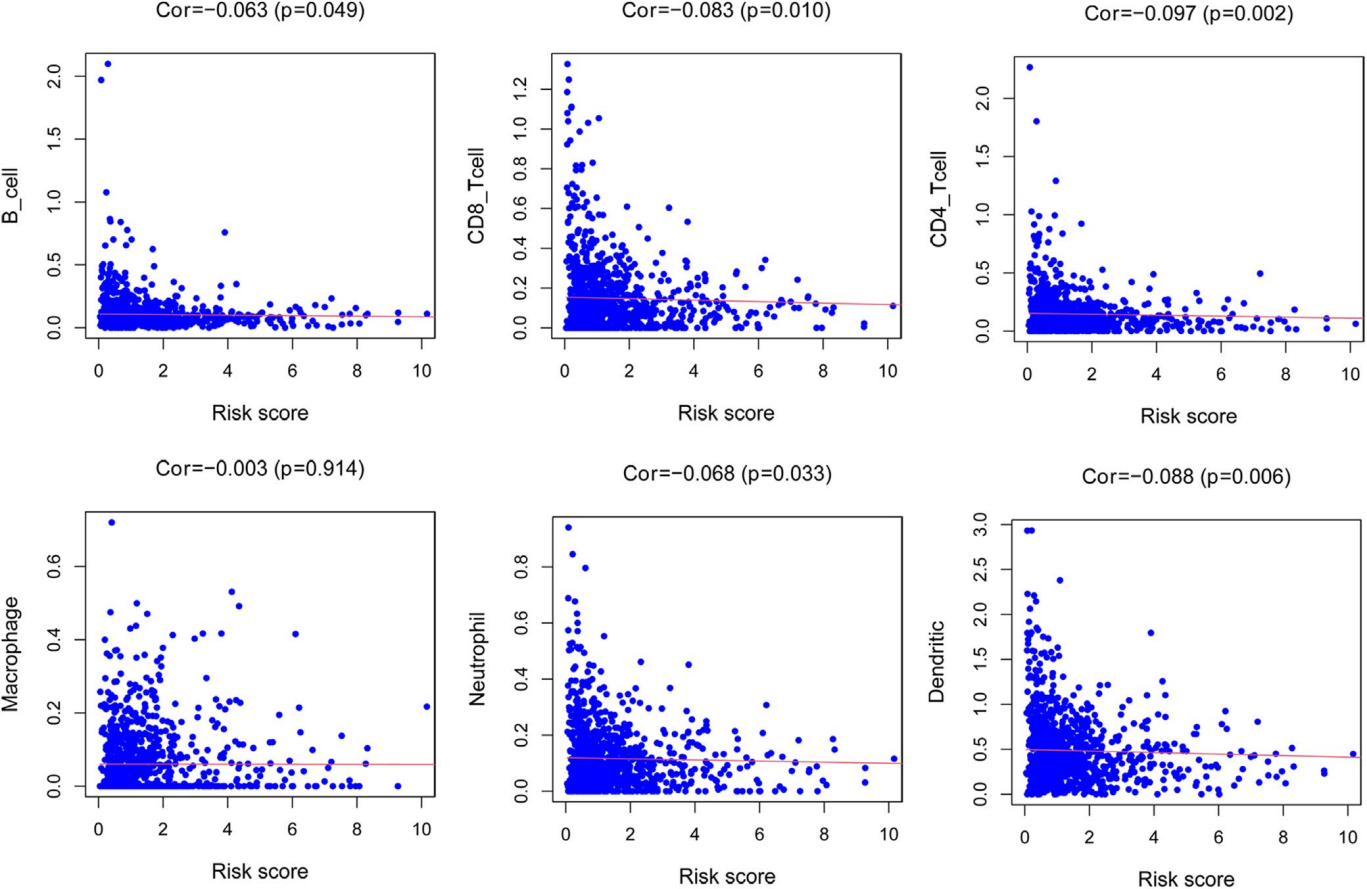
The correlations of B cell, CD4+ T cells, Macrophages, Neutrophils, and DCs

The risk score of ferritinophagy prognostic model is negatively correlated with B cells (*r* = −0.063, *p* = 0.049), CD8+ T cells (*r* = −0.083, *p* = 0.010), CD4+ T cells (*r* = −0.097, *p* = 0.002), neutrophils (*r* = −0.068, *p* = 0.033), and dendritic cells (*r* = 0.088, *p* = 0.006) (Figure 7).

## 4 DISCUSSION

Although numerous studies^10^, ^11^, ^12^, ^13^ have investigated the genes that might regulate cancer cell death through ferroptosis, their correlations with prognosis remain worth exploring. In this article, we explored the expression levels of 259 ferritinophagy‐related genes in breast cancer. The Cox univariate analysis was conducted to determine the relationships between ferroptosis and the prognosis. The prognostic model was constructed according to the prognostic genes. We identified 38 ferritinophagy‐related prognostic genes in total.

Breast cancer is a common disease, and its treatment is based on molecular subtypes. Compared with other breast cancer subtypes, triple‐negative breast cancer is highly invasive. Patients with triple‐negative breast cancer are more likely to die, accounting for approximately 40% of mortality rates within the first 5 years after being diagnosed.^14^ In the TCGA database, there were 134 triple‐negative breast cancer patients, and 133 patients participated in follow‐ups for at least 1 month. The forest plot indicated that 12 genes showed a significant correlation with a poor prognosis, while 9 genes showed a significant correlation with a better prognosis. Nitric oxide synthase (NOS2) has been reported in previous studies^15^, ^16^, ^17^ to be related to poor survivability in breast cancer estrogen receptor‐negative breast cancer patients. We have found that TGFBR1 is crucial in TGF‐beta signaling and it plays a dual role in cancer,^18^ and TGFBR1 protein expressions are associated with a significantly increased risk of breast cancer^19^ (Figure S1). Amplifying HER‐2/neu accounted for approximately 20% of all breast cancers.^20^, ^21^ Although trastuzumab, pertuzumab, and lapatinib have been proven as targeted drugs for HER‐2‐positive breast cancer, overexpression of HER‐2 was associated with worse survival rates.^22^ Therefore, exploring the hub gene is significant in HER‐2‐positive breast cancer. Nuclear receptor coactivator 4 (NCOA4) is crucial in the autophagic degradation of ferritin.^23^ The overexpression of NCOA4 induced ferritin by regulating the accumulation of intracellularly free iron and glutathione.^24^ In MCF7 breast cancer cells, NCOA4 was associated with proliferation and invasion.^25^ In our analysis, NCOA4 is related to poor survivability in HER‐2‐positive breast cancer. Thioredoxin reductase 1 (TXNRD1) plays dual roles, because it can induce cancer and inhibit cancer. It can protect normal cells from carcinogenesis in normal tissue, while also promoting cancer progression in cancer tissue.^26^ The overexpression of TXNRD1 was proved to be associated with poor prognosis in breast cancer through an increase in oxidative stresses.^27^ In our analysis, 16 genes were associated with poor prognosis while 7 genes were associated with better prognosis in HER‐2‐positive breast cancer (Figure S2). Luminal breast cancer accounted for approximately 60%–70% of all breast cancer.^28^, ^29^ Endocrine therapy is the main treatment for luminal breast cancer, and current guidelines suggested that luminal A patients could be solely treated with endocrine therapy. Patients with luminal A breast cancer had a better prognosis than other breast cancer subtypes. There are 14 genes associated with poor prognosis and 21 genes associated with better prognosis in luminal A breast cancer (Figure S3). Luminal B breast cancer is aggressive and has a prognosis similar to triple‐negative or HER‐2‐positive breast cancer.^30^ Li et al.^31^ reported that luminal B subtypes occurred in 48.1% of recurrences and metastasis samples. Despite the variety in treatments, it was still difficult to cure luminal B subtypes. We discovered 11 genes associated with the aggressiveness of luminal B subtypes (Figure S4). These are promising prognostic biomarkers in luminal B subtypes.

Immune surveillance and cancer biology are intertwined.^32^, ^33^ The tumor microenvironment has become a focus. In the ferritinophagy prognostic model, infiltrating levels of B cells, CD8+ T cells, CD4+ T cells, neutrophils, and dendritic cells are correlated with the risk scores. Ferroptosis was known to affect breast cancer cells by inhibiting their proliferation, mitigating their resistance to chemotherapy drugs, and enhancing their sensitivity to radiotherapy.^9^, ^34^ We investigated the potential prognostic biomarkers in breast cancer to offer further information. However, we were limited in exploring ferroptosis mechanisms and their potential role in breast cancer treatment. In our study, all breast cancer information was obtained from the TCGA database, and the patients are primarily Americans. Breast cancer patients from other regions remain to be further verified with additional evidence.

## 5 CONCLUSIONS

In summary, this present study conducted a bioinformatics analysis based on the TCGA database and established a prognostic model for 259 ferritinophagy‐related genes. There were significant differences between subtypes, so we analyzed different molecular subtypes. In this study, prognostic biomarkers were found in different subtypes. The underlying mechanisms of these ferritinophagy‐related genes could be addressed in the future.

## CONFLICT OF INTEREST

The authors declare that they have no conflicts of interest.

## AUTHOR CONTRIBUTION

Yanlin Gu involved in conceptualization. Xiaohua Li involved in data curation. Yanlin Gu and Qi Zhu involved in formal analysis. Xiaohua Li and Liyan Jin involved in investigation. Guoqin Jiang involved in methodology. Qi Zhu involved in project administration. Liyan Jin and Guoqin Jiang involved in supervision. Yanlin Gu wrote the original draft. Liyan Jin, Guoqin Jiang involved in writing, reviewing the editing the article.

## CONSENT FOR PUBLICATION

The authors confirm that the work described has not been published before. Its publication has been approved by all co‐authors.

## Supporting information

Figure S1

Figure S2

Figure S3

Figure S4

## Data Availability

All data generated or analyzed during this study could be obtained from TCGA database.
