# Supplementary figures and images for: The role of ferroptosis‐related genes for overall survival prediction in breast cancer
Source: J Clin Lab Anal. 2021 Nov 6;35(12):e24094. doi: 10.1002/jcla.24094 (PMC8649350; doi:10.1002/jcla.24094)

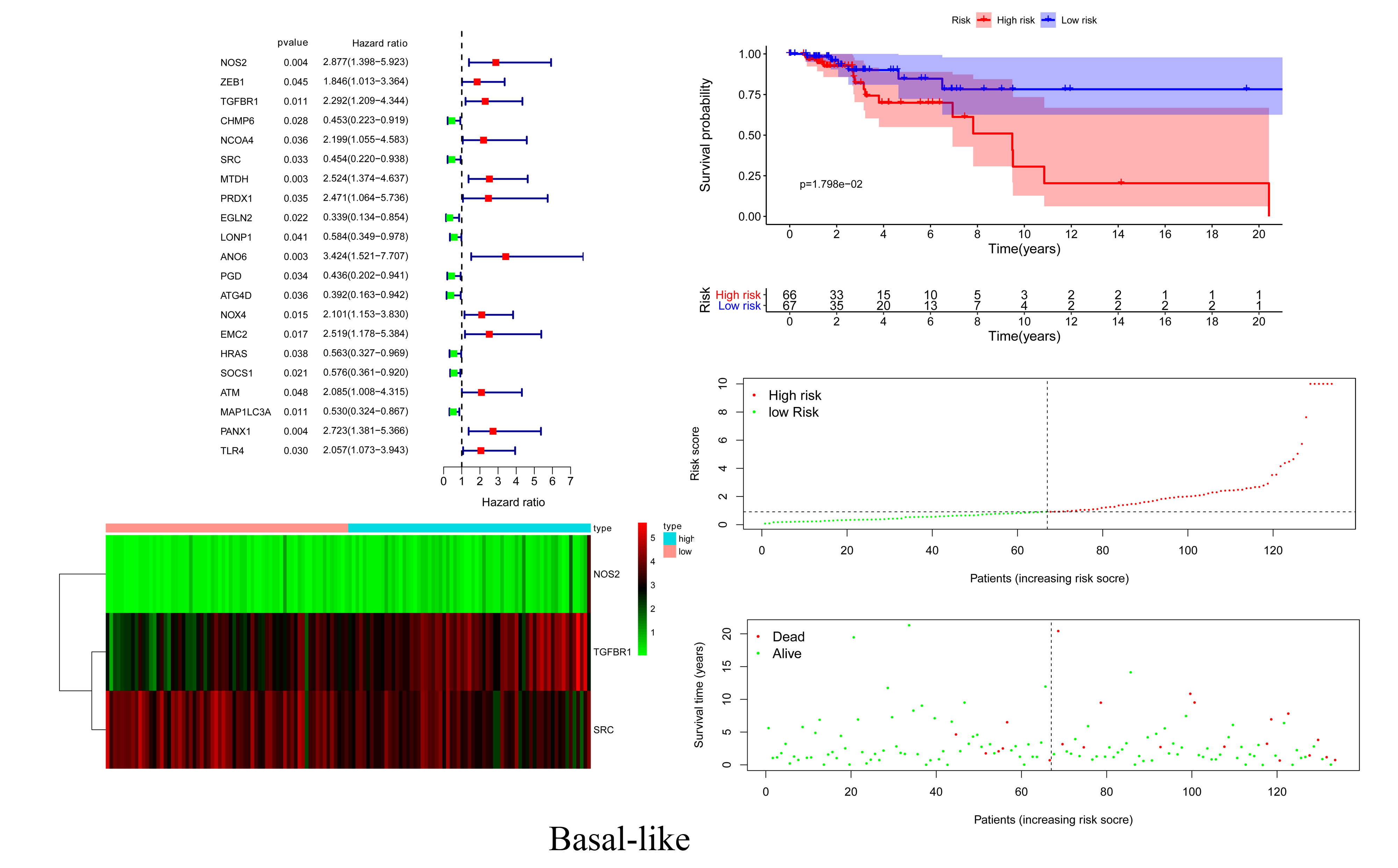

Supplement: Supplementary file 1 — Figure S1 [file JCLA-35-e24094-s001.tif]

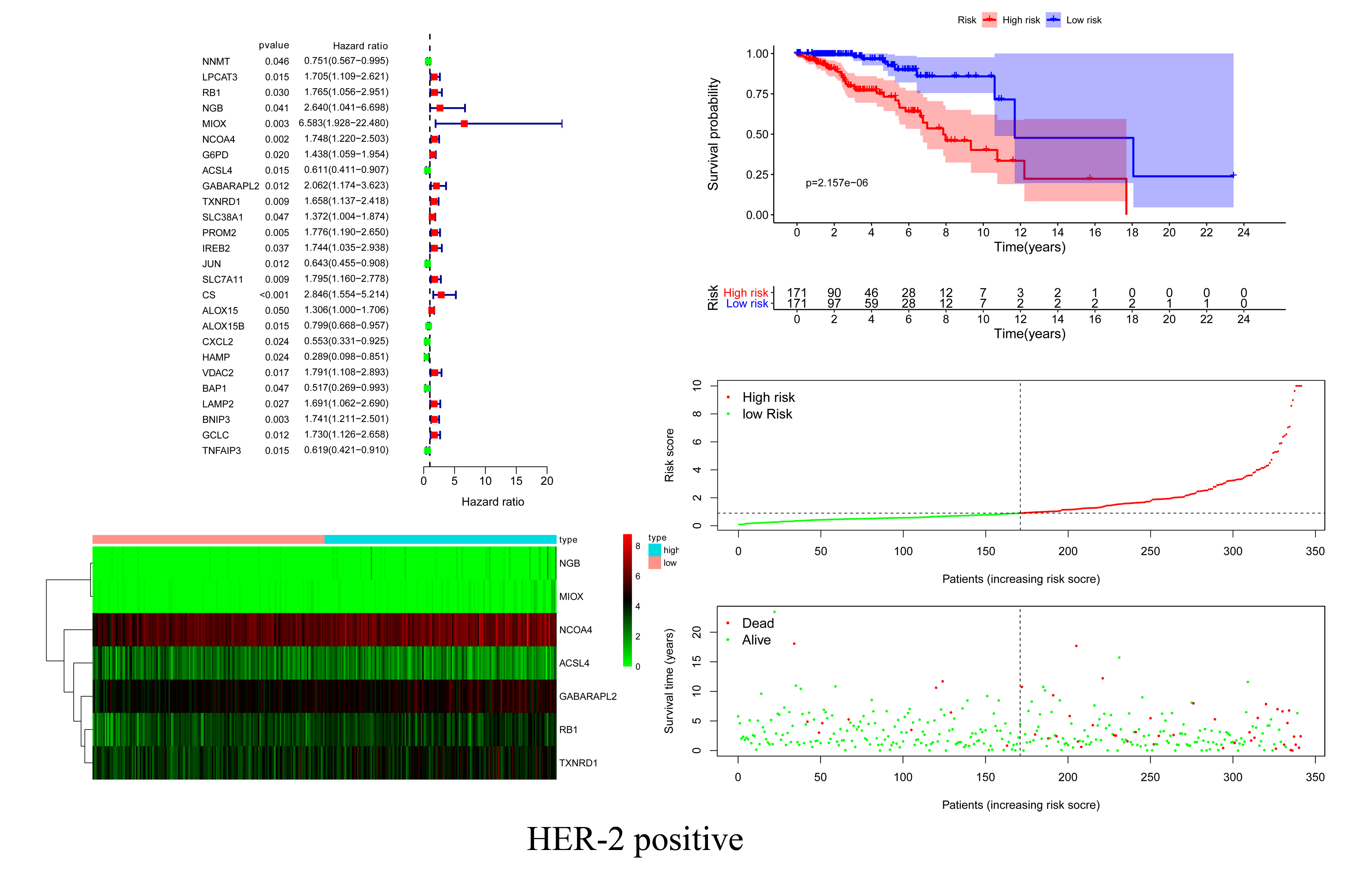

Supplement: Supplementary file 2 — Figure S2 [file JCLA-35-e24094-s003.tif]

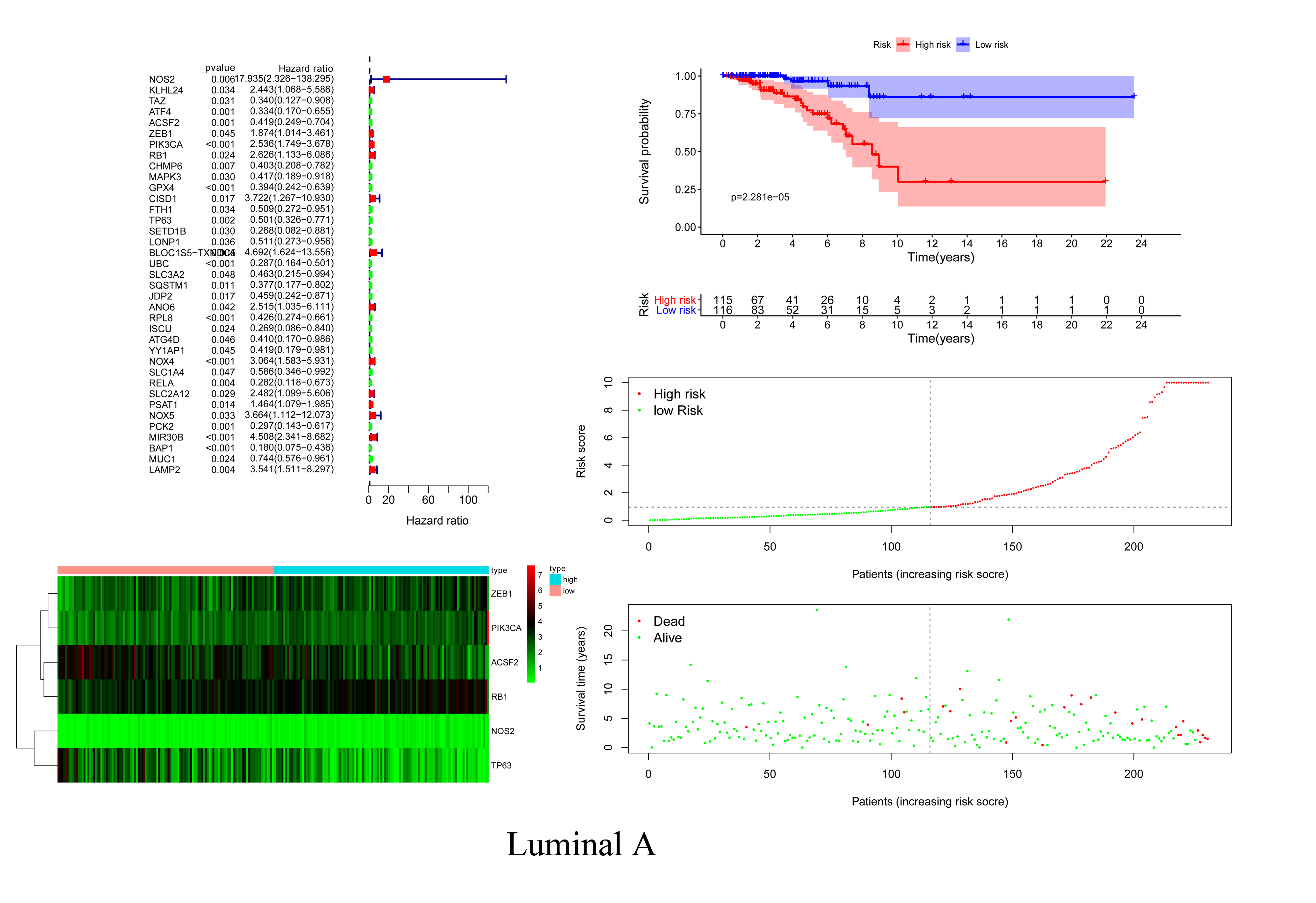

Supplement: Supplementary file 3 — Figure S3 [file JCLA-35-e24094-s002.tif]

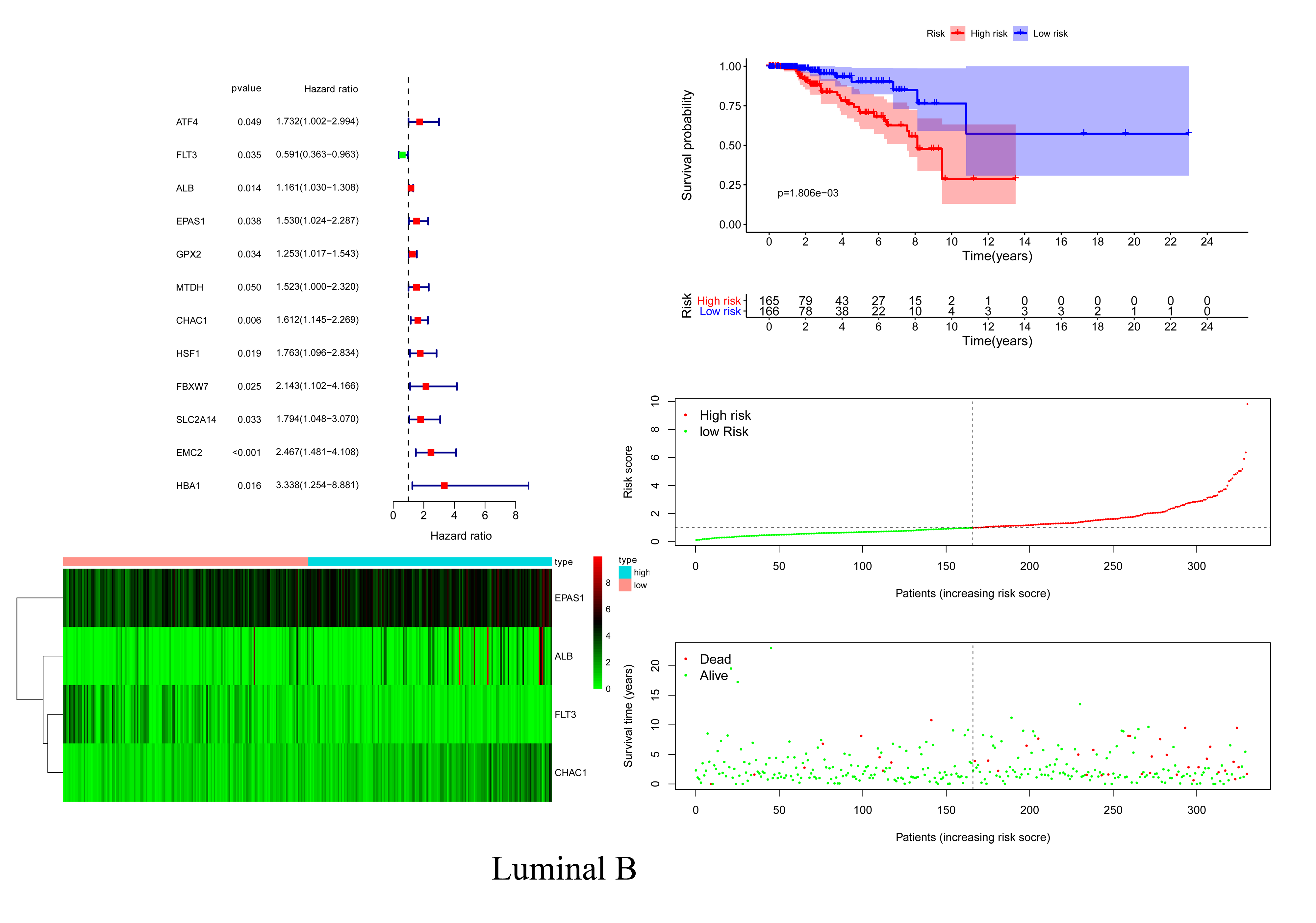

Supplement: Supplementary file 4 — Figure S4 [file JCLA-35-e24094-s004.tif]
